# Supplementary figures and images for: Integrated experimental-computational analysis of a HepaRG liver-islet microphysiological system for human-centric diabetes research
Source: PLoS Comput Biol. 2022 Oct 19;18(10):e1010587. doi: 10.1371/journal.pcbi.1010587 (PMC9621595; doi:10.1371/journal.pcbi.1010587)

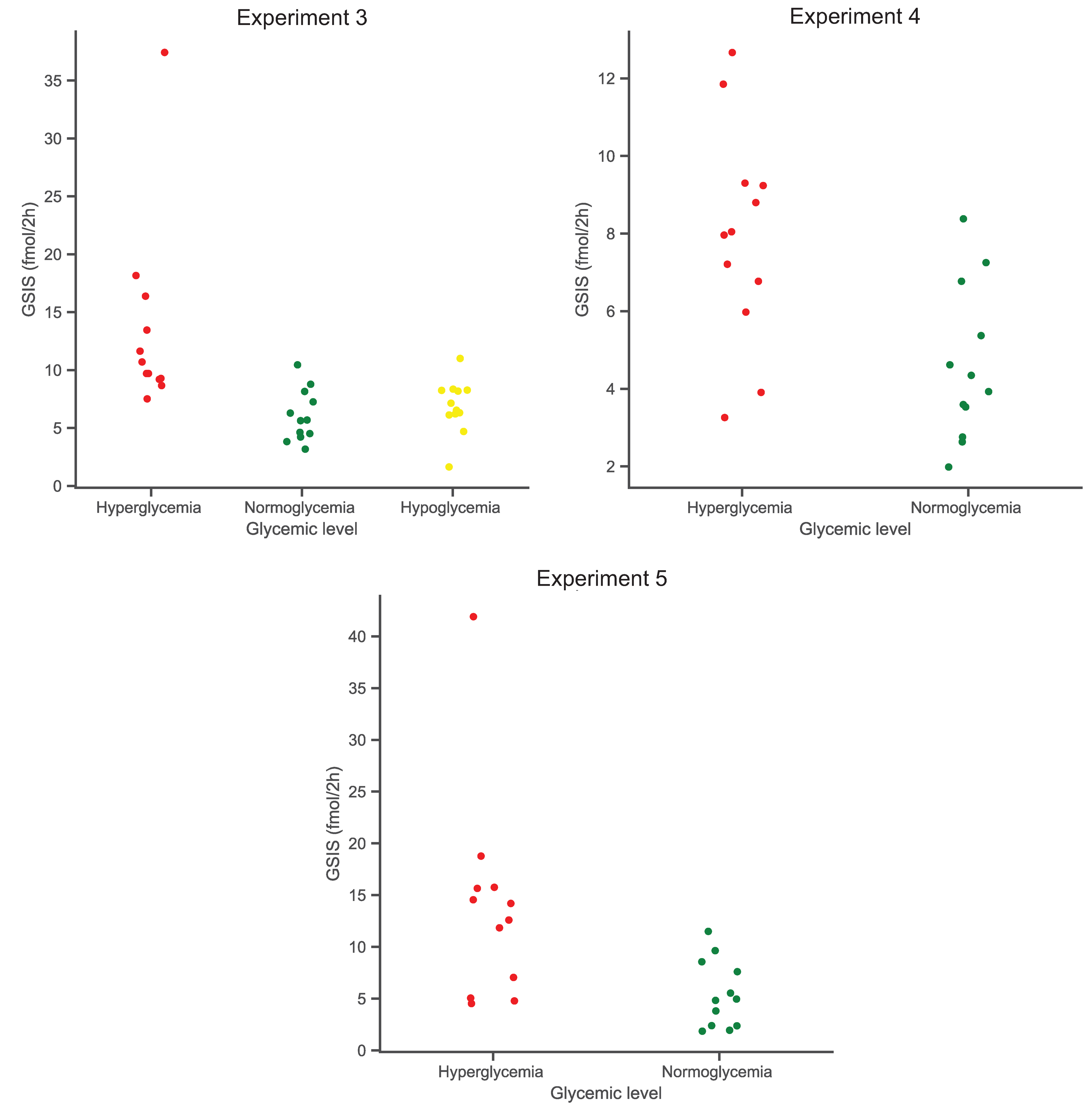

Supplement: S1 Fig — The pancreatic islets were collected from the MPS after 15 days of co-culture. During the co-culture, they were exposed to either hyper- (11mM, red), normo- (5.5 mM, green) or hypoglycemic conditions (2.8 mM, yellow). At day 13, a GTT with a glucose load of 11 mM was performed in all co-cultures. After being collected from the MPS, the pancreatic islets were incubated in low glucose (2.8 mM) over 2h, following 2h incubation in high glucose (16.8 mM). The results correspond to experiments 3 (A), 4 (B) and 5 (C). (TIF) [file pcbi.1010587.s001.tif]

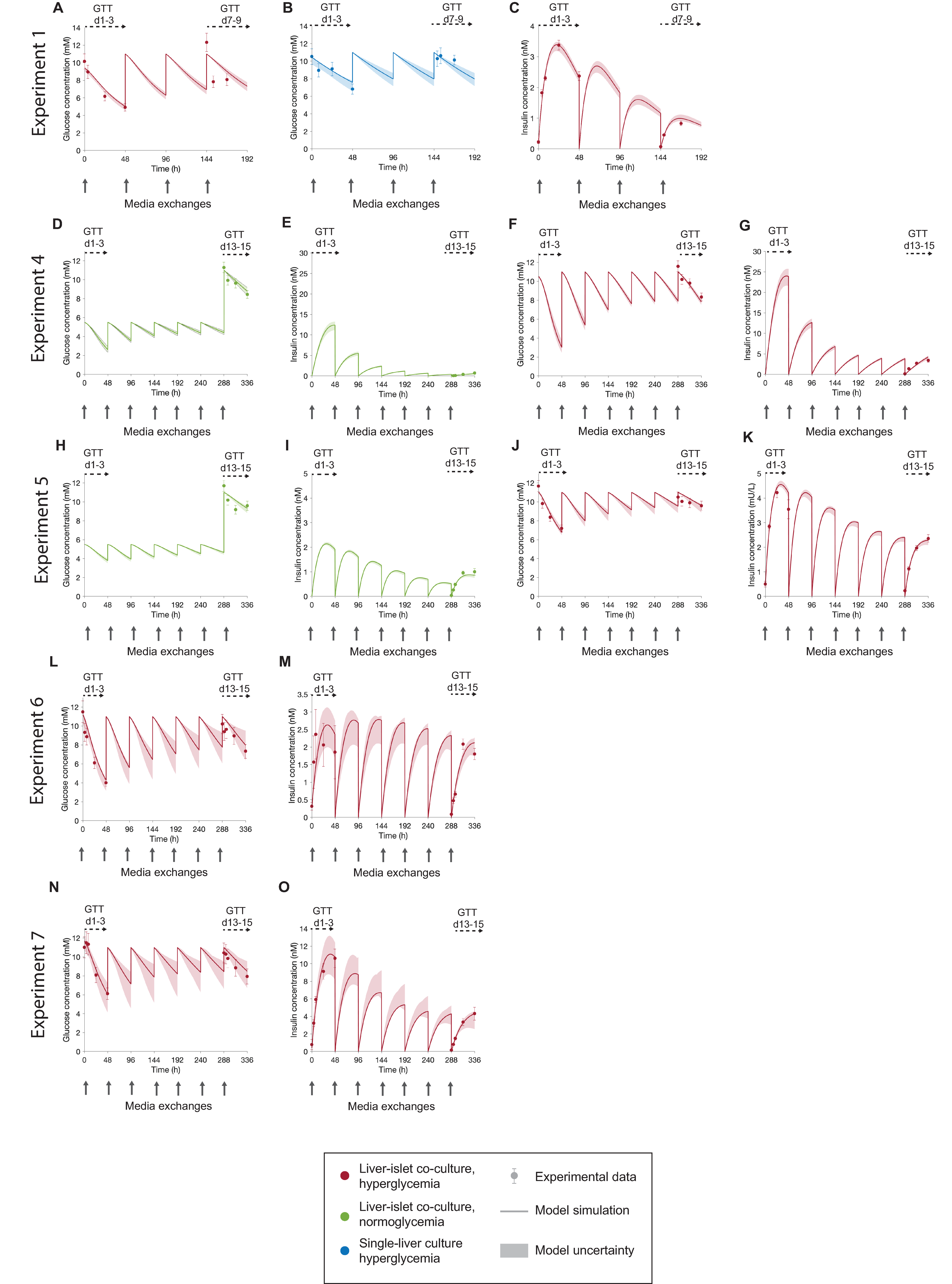

Supplement: S2 Fig — Experiment 1 (A-C): Glucose concentration in the liver-islet co-culture under hyperglycemia (A), glucose concentration in the single-liver culture under hyperglycemia (B), insulin concentration in the liver-islet co-culture under hyperglycemia (C); experiment 4 (D-G): glucose concentration in the liver-islet co-cultures under normoglycemia (D), insulin concentration in the liver-islet co-culture under normoglycemia (E), glucose concentration in the liver-islet co-culture under hyperglycemia (F), insulin concentration in the liver-islet co-culture under hyperglycemia (G); experiment 5 (H-K): glucose concentration in the liver-islet co-culture under normoglycemia (H), insulin concentration in the liver-islet co-culture under normoglycemia (I), glucose concentration in the liver-islet co-culture under hyperglycemia (J), insulin concentration in the liver-islet co-culture under hyperglycemia (K); experiment 6 (L-M): Glucose concentration in the liver-islet co-culture under normoglycemia (L), insulin concentration in the liver-islet co-culture under normoglycemia (M); experiment 7 (N-O): Glucose concentration in the liver-islet co-culture under hyperglycemia (N), insulin concentration in the liver-islet co-culture under hyperglycemia (O). In hyperglycemic and normoglycemic conditions, co-cultures were exposed to 11 mM or 5.5 mM glucose in each media exchange (arrows), respectively. Model uncertainty is shown as shaded areas in panels A-O. Data in panels A-O are presented as mean ± SEM, where the number of replicas considered for each experiment are: n = 4 (experiments 1, 6 and 7 for all glycemic conditions), n = 5 (experiment 4 for all glycemic conditions and experiment 5 for hyperglycemia) and n = 10 for experiment 5 under normoglycemia. (TIF) [file pcbi.1010587.s002.tif]

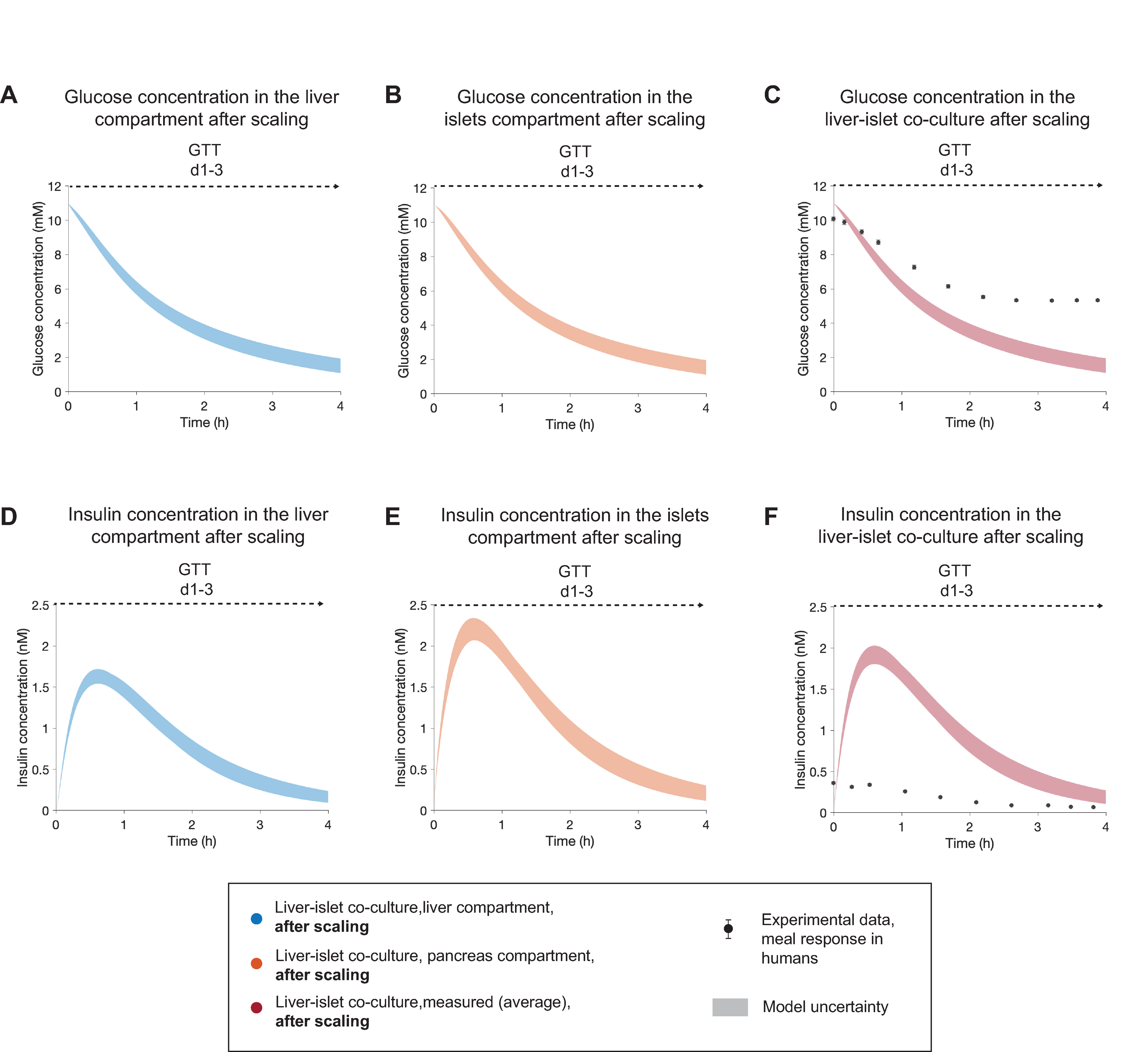

Supplement: S3 Fig — The results correspond to a single experiment (experiment 1). A,B,D,E: Model predictions of glucose (A,B) and insulin (D,E) in the liver and pancreas compartments after scaling. C shows the comparison between the model prediction of plasma glucose concentration after scaling and experimental data of glucose response to a meal in healthy subjects [66]. The model-based prediction of the insulin response and the experimental measurements of insulin are compared in F. The predictions are computed for the GTT initiated at day 1 (GTT d1-3). The experimental data were acquired in a group of 204 normal subjects [66]. We consider the time point of peak glucose concentration in the experimental data as time = 0 h for this study, since the MPS lacks an intestinal compartment and glucose is administered directly to both the liver and pancreas compartments. Data are presented as mean ± SEM (n = 204). Model uncertainty is depicted as shaded areas in (A-F). (TIF) [file pcbi.1010587.s003.tif]

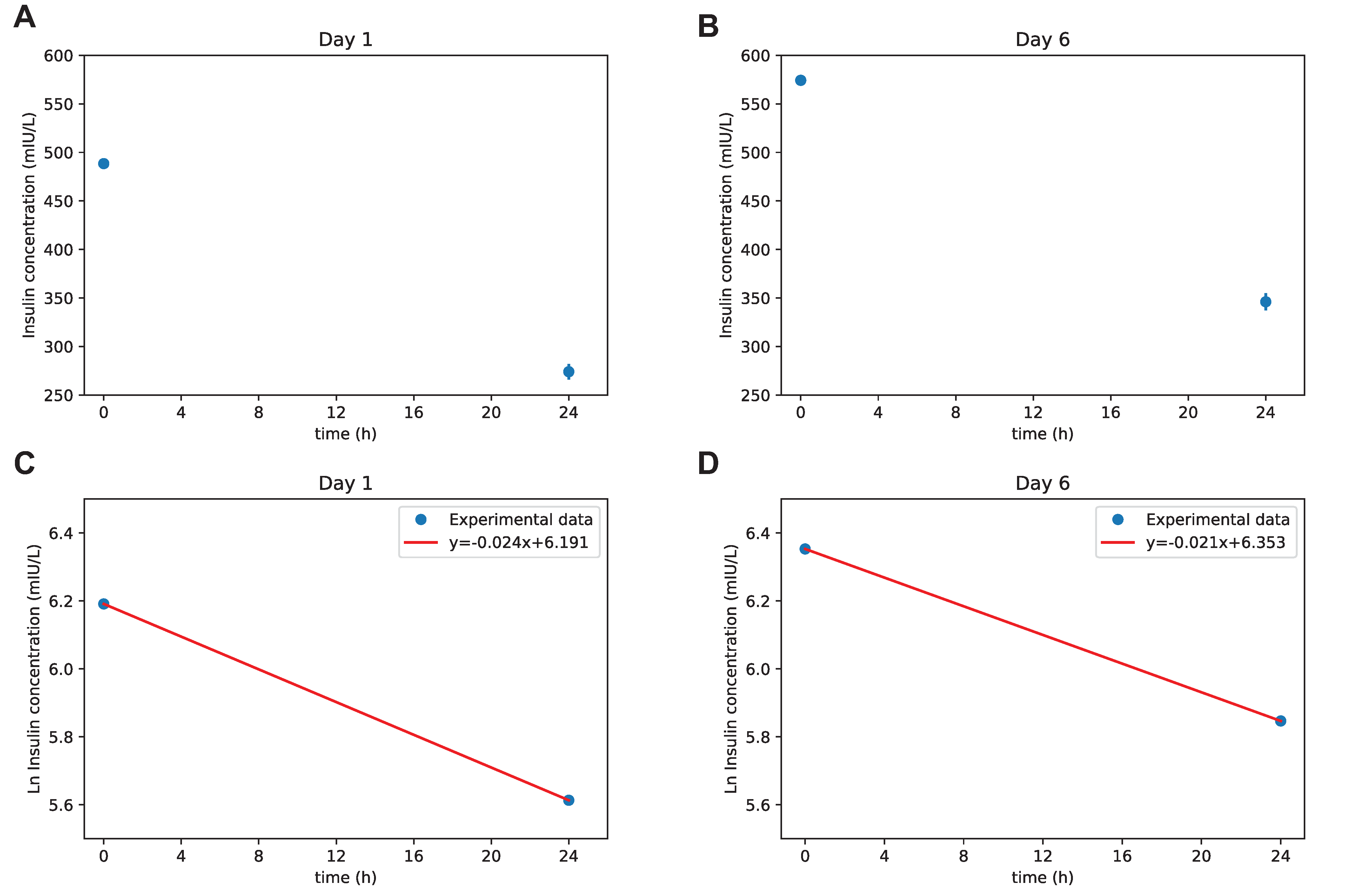

Supplement: S4 Fig — The experimental data corresponds to a single experiment, where single-liver cultures were exposed to hyperglycemic conditions (11 mM glucose in each co-culture medium exchange during a 7-day co-culture period). At days 1 and 6, an insulin dose was added to the co-culture medium to assess insulin clearance. Data in (A,B) are presented as mean ± SEM (n = 4). In (C, D), a linear regression model was fitted to the mean values of the experimental data for each day to estimate the hepatic insulin elimination rate constant. The resulting estimated values are kday1 = 0.024 (1/h) and kday7 = 0.021 (1/h) for days 1 and 7, respectively. (TIF) [file pcbi.1010587.s004.tif]

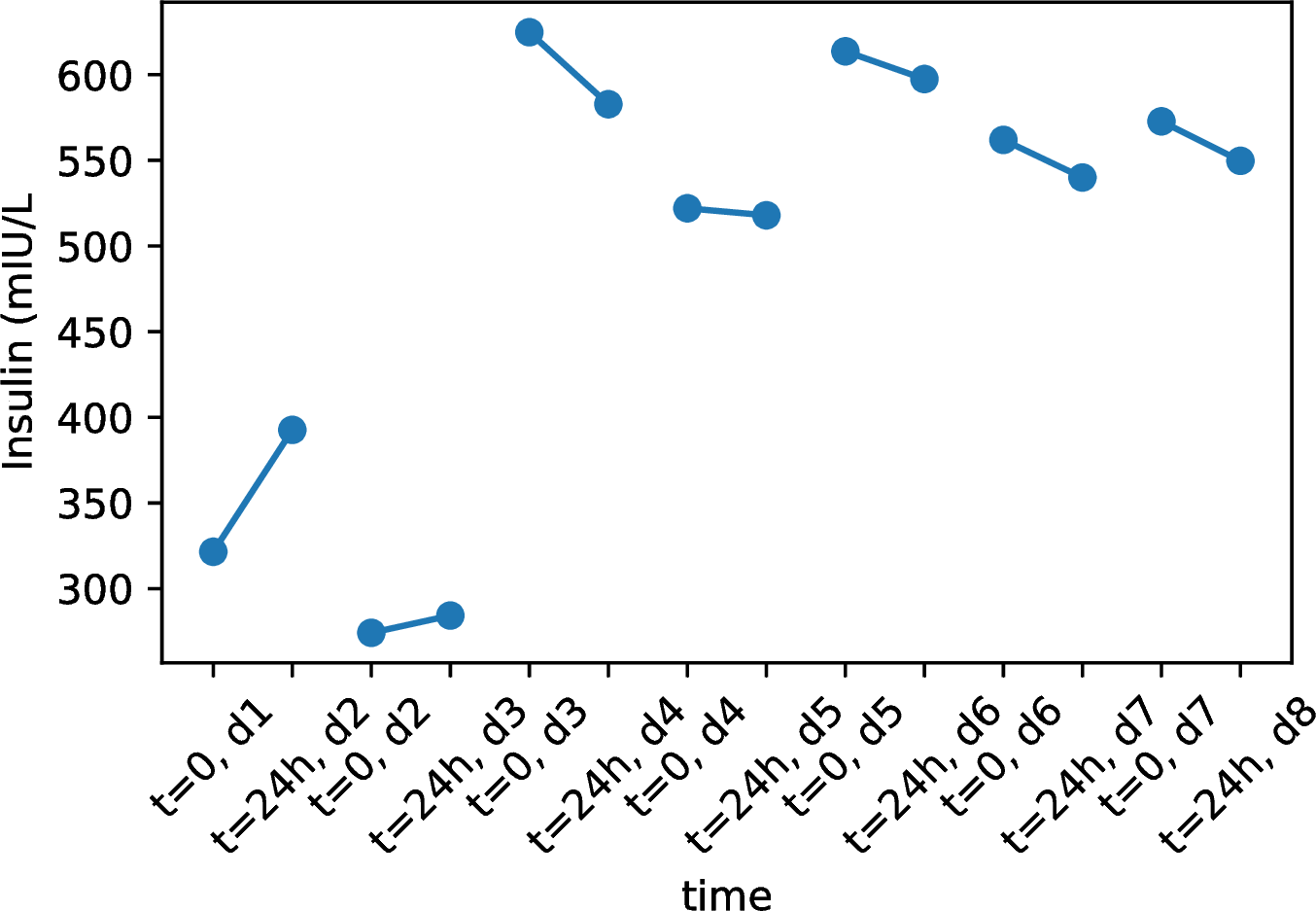

Supplement: S5 Fig — Media exchanges were performed every 24 hours, and samples of the culture media were taken directly after each media exchange (t = 0 h) as well as 24 hours after (t = 24 h). In each media exchange, a specific amount of insulin was added to the culture medium. Data correspond to a single chip replicate (n = 1). (TIF) [file pcbi.1010587.s005.tif]
