# Supplementary material for: Integrated experimental-computational analysis of a HepaRG liver-islet microphysiological system for human-centric diabetes research
Source: PLoS Comput Biol. 2022 Oct 19;18(10):e1010587. doi: 10.1371/journal.pcbi.1010587 (PMC9621595; doi:10.1371/journal.pcbi.1010587)
Supplement: S1 Table — GTT: Glucose tolerance test. (PDF) [file pcbi.1010587.s006.pdf]

S1 Table: Summary of the experimental settings used in the MPS experiments and the measurements acquired for calibration and evaluation of the experiment-specific computational models. GTT: Glucose tolerance test.

**Experiment number**	**Experiment arms**	**Length of co-culture**	**Glycemic regimes**	**Time of GTTs**	**Measurements for the experiment-specific computational model**
1[1]	Single-liver<br>Liver-islet	9 days	Hyperglycemia	Single-liver: Days 1-3, Days 7-9<br>Liver-islet: Days 1-3, Days 7-9	**Single-liver:** Glucose concentration during GTTs (calibration)<br>**Liver-islet:** Glucose and insulin concentrations during GTTs (calibration)
2[1]	Single-liver<br>Liver-islet	15 days	Hyperglycemia	Single-liver: Days 1-3, Days 13-15<br>Liver-islet: Days 1-3, Days 13-15	**Single-liver:** Glucose concentration during GTTs (calibration)<br>**Liver-islet:** Glucose and insulin concentrations during GTTs (calibration)<br>Glucose and insulin concentrations at both liver and pancreas compartments measured every 48 h between days 3 and 13 of the co-culture (evaluation)
3	Liver-islet	15 days	Hyperglycemia	Hyperglycemia: Days 1-3, Days 13-15	Glucose and insulin concentrations during GTTs for hyper-and normo-glycemia (calibration)

[1] Experiments included in (3)

			Normoglycemia<br>Hypoglycemia	Normoglycemia: Days 13-15<br>Hypoglycemia: Days 13-15	Glucose and insulin concentrations during GTTs for hypoglycemia (evaluation)
4	Liver-islet	15 days	Hyperglycemia<br>Normoglycemia	Days 13-15	Glucose and insulin concentrations during GTTs for all glycemic regimes (calibration)
5	Liver-islet	15 days	Hyperglycemia<br>Normoglycemia	Hyperglycemia: Days 1-3,Days 13-15<br>Normoglycemia: Days 13-15	Glucose and insulin concentrations during GTTs for all glycemic regimes (calibration)
6	Liver-islet	15 days	Hyperglycemia	Days 1-3, Days 13-15	Glucose and insulin concentrations during GTTs
7	Liver-islet	15 days	Hyperglycemia	Days 1-3, Days 13-15	Glucose and insulin concentrations during GTTs
