# Supplementary material for: Integrated experimental-computational analysis of a HepaRG liver-islet microphysiological system for human-centric diabetes research
Source: PLoS Comput Biol. 2022 Oct 19;18(10):e1010587. doi: 10.1371/journal.pcbi.1010587 (PMC9621595; doi:10.1371/journal.pcbi.1010587)
Supplement: S2 Table — Acceptable models could be inferred for every experiment. The threshold for the χ2 test with 95% significance is calculated based on the number of data points in the experimental time-series for each experiment. (PDF) [file pcbi.1010587.s007.pdf]

S2 Table: Summary of model evaluations for all the MPS experiments included in the analysis: Acceptable models could be inferred for every experiment. The threshold for the χ^2 test with 95% significance is calculated based on the number of data points in the experimental time-series for each experiment.

Experiment number	Agreement with experimental data (χ^2 error, threshold)
1	29.45 (<31.41)
2	21.62 (<31.41)
3	7.55 (<37.65)
4	17.48 (<26.27)
5	28.37 (<37.65)
6	20.21 (<31.41)
7	7.01 (<31.41)
