# Supplementary material for: Integrated experimental-computational analysis of a HepaRG liver-islet microphysiological system for human-centric diabetes research
Source: PLoS Comput Biol. 2022 Oct 19;18(10):e1010587. doi: 10.1371/journal.pcbi.1010587 (PMC9621595; doi:10.1371/journal.pcbi.1010587)
Supplement: S3 Table — Values indicated as (-) were not estimated because there were no available data to perform the estimation. (PDF) [file pcbi.1010587.s008.pdf]

S3 Table: Estimated values of the experiment-specific parameters for each MPS experiment included in the study. Values indicated as (-) were not estimated because there were no available data to perform the estimation.

**Parameter**	**Unit**	**Estimated value for each MPS experiment**						
		**1**	**2**	**3**	**4**	**5**	**6**	**7**
E_{G0}	1/h	1.19	1.02	1.05	0.75	0.36	0.83	0.84
$CL_{I,spheroids}$	1/h	17.35	15.27	22.57	1.24	11.77	7.04	6.55
S_{I0}	L/mIU/h	$3.12 \cdot 10^{-3}$	$2.35 \cdot 10^{-3}$	$1.08 \cdot 10^{-2}$	$1.63 \cdot 10^{-3}$	$4.33 \cdot 10^{-3}$	$1.68 \cdot 10^{-2}$	$2.79 \cdot 10^{-3}$
σ_{max}	mIU/L/h	$6.85 \cdot 10^{6}$	$1.06 \cdot 10^{7}$	$5.31 \cdot 10^{6}$	$1.70 \cdot 10^{7}$	$5.60 \cdot 10^{6}$	$2.64 \cdot 10^{6}$	$9.68 \cdot 10^{6}$
α	h^2	87.76	218.87	194.42	48.07	157.89	383.92	107.54
k_v		1.93	4.89	13.60	26.97	10.20	1	16.46
$I_{max,Si}$		$1.81 \cdot 10^{-3}$	0.49	1	0.53	0.99	0.98	0.97
$EC50_{Si}$	mmol·h/L	4621.79	160.69	100.1	138.55	195.61	100	100.2
ΔG_{d1}	mmol/L	-1.78	-1.48	-	-0.5	-0.04	-0.78	0.62
ΔG_{d13}	mmol/L	-	-1.55	-	0.42	-0.28	-0.94	-0.67
ΔI_{d1}	mIU/L	34.49	34.38	-	-	90.95	49.94	160.64
ΔI_{d13}	mIU/L	-	0	-	21.71	32.63	10.85	23.61
