# Supplementary material for: Integrated experimental-computational analysis of a HepaRG liver-islet microphysiological system for human-centric diabetes research
Source: PLoS Comput Biol. 2022 Oct 19;18(10):e1010587. doi: 10.1371/journal.pcbi.1010587 (PMC9621595; doi:10.1371/journal.pcbi.1010587)
Supplement: S1 Appendix — Supplementary description of the computational model, and comparison of hepatic insulin clearance rates in the HepaRG liver-islet MPS with human in situ values. (PDF) [file pcbi.1010587.s009.pdf]

S1 Appendix to

Integrated experimental-computational analysis of a HepaRG liver-islet microphysiological system for human-centric diabetes research

Belén Casas[1,2], Liisa Vilén[1], Sophie Bauer[3], Kajsa P. Kanebratt[1], Charlotte Wennberg Huldt[4], Lisa Magnusson[4], Uwe Marx[3], Tommy B. Andersson[1], Peter Gennemark[1,2,&], Gunnar Cedersund[2,5,*,&]

1 Drug Metabolism and Pharmacokinetics, Research and Early Development, Cardiovascular, Renal and Metabolism (CVRM), BioPharmaceuticals R&D, AstraZeneca, Gothenburg, Sweden.

2 Department of Biomedical Engineering, Linköping University, Linköping, Sweden.

3 TissUse GmbH, Berlin, Germany.

4 Bioscience, Research and Early Development, Cardiovascular, Renal and Metabolism (CVRM), BioPharmaceuticals R&D, AstraZeneca, Gothenburg, Sweden.

5 Center for Medical Image Science and Visualization (CMIV), Linköping University, Linköping, Sweden.

[&]Shared senior author

*Corresponding author

Gunnar Cedersund, Department of Biomedical Engineering
Linköping University,
SE-581 83 Linköping, Sweden.
E-mail: gunnar.cedersund@liu.se

Supplementary methods

1. Supplementary description of the computational model: model equations

The computational model developed in this study is formulated with the set of ordinary differential equations (ODEs) described below. A detailed description of the key equations in the model is given in the main article (Methods, Section 2.2).

Glucose dynamics in the liver compartment

Glucose in the co-culture medium of the liver compartment is determined by the dose of glucose, glucose production and uptake from the HepaRG/HHSteC spheroids, and glucose exchange with the pancreas compartment:

$$\frac{dNG_{m,liver}(t)}{dt} = G_d(t) + V_{HepaRG,spheroids} \cdot EGP(t) - V_{HepaRG,spheroids}\left(E_{G0} + S_I(t) \cdot \frac{NI_{m,liver}(t)}{V_{m,liver}}\right)\frac{NG_{m,liver}(t)}{V_{m,liver}} + Q \cdot \frac{NG_{m,pancreas}(t)}{V_{m,pancreas}} - Q \cdot \frac{NG_{m,liver}(t)}{V_{m,liver}} \ (mmol/h) \qquad \text{(S 1)}$$

$NG_{m,liver}(t)$: Number of glucose molecules in the liver compartment (mmol)

$NG_{m,pancreas}(t)$: Number of glucose molecules in the pancreas compartment (mmol)

$NI_{m,liver}(t)$: Number of insulin molecules in the liver compartment (mIU)

$G_d(t)$: Glucose input rate to the liver and pancreas compartments, determined by glucose variations because of media exchanges. Co-culture medium with 11 mM, 5.5 mM or 2.8 mM glucose is added into each of the culture compartments in each media exchange for the hyper-, normo- and hypoglycemic regimes, respectively.

E_{G0}: Insulin-independent glucose disposal rate of the HepaRG/HHSteC spheroids (1/h)

$EGP(t)$: Endogenous glucose production from the HepaRG/HHSteC spheroids (mmol/L/h)

Q: Flow rate between culture compartments (L/h)

$V_{HepaRG,spheroids}$: Volume of HepaRG cells in the HepaRG/HHSteC spheroids (L)

$V_{m.liver}$: Volume of co-culture media in the liver compartment (L)

$V_{m.pancreas}$: Volume of co-culture media in the pancreas compartment (L)

The insulin sensitivity of the HepaRG/HHSteC spheroids, $S_I(t)$, is described as follows:

$$S_I(t) = S_{I0} \cdot \left(1 - \frac{I_{max,Si} \cdot G_{int}(t)}{EC50_{Si} + G_{int}(t)}\right) \ (L/mIU/h)$$

(S 2)

S_{I0}: Insulin sensitivity of the HepaRG/HHSteC spheroids at the start of the co-culture (L/mIU/h)

$I_{max,Si}$: Maximal fractional reduction of insulin sensitivity

$EC50_{Si}$: Value of time integral of excess glucose (i.e. difference between glucose levels in the co-culture media and the normoglycemic concentration of 5.5 mM) providing half of $I_{max,Si}$ (mmol·h/L)

The variable $G_{int}(t)$ increases progressively as the HepaRG/HHSteC spheroids are exposed to glucose levels above the normoglycemic range $\left(\frac{NG_{m,liver}(t)}{V_{m,liver}} - Gnormo \geq 0\right)$, as given by:

$$\frac{dG_{int}(t)}{dt} = \begin{cases} \frac{NG_{m,liver}(t)}{V_{m,liver}} - G_{normo} & \frac{NG_{m,liver}(t)}{V_{m,liver}} - Gnormo \geq 0 \\ 0 & \frac{NG_{m,liver}(t)}{V_{m,liver}} - Gnormo < 0 \end{cases} \ (\frac{mmol}{L})$$

(S 3)

G_{normo}: Glucose concentration considered for normoglycemia (5.5 mM)

Glucose dynamics in the pancreas compartment

Glucose content in the pancreas compartment is described as:

$$\frac{dNG_{m,pancreas}(t)}{dt} = G_d(t) + \ Q \cdot \frac{NG_{m,liver}(t)}{V_{m,liver}} - Q \cdot \frac{NG_{m,pancreas}(t)}{V_{m,pancreas}} \ (mmol/h)$$

(S 4)

Insulin dynamics in the liver compartment

Insulin content in the liver compartment is described by the following equation:

$$\frac{dNI_{m,liver}(t)}{dt} = Q \cdot \frac{NI_{m,pancreas}(t)}{V_{m,pancreas}} - Q \cdot \frac{NI_{m,liver}(t)}{V_{m,liver}} - V_{HepaRG,spheroids} \cdot CL_{I,spheroids} \cdot \frac{NI_{m,liver}(t)}{V_{m,liver}} \; (mIU/h) \qquad \text{(S 5)}$$

$CL_{I,spheroids}$: Insulin clearance by the HepaRG/HHSteC spheroids (1/h)

Insulin dynamics in the pancreas compartment

Insulin content in the pancreas compartment is determined by the release of insulin from the β cells in the pancreatic islets:

$$\frac{dNI_{m,pancreas}(t)}{dt} = V_{\beta,islets}(t) \cdot \sigma(t) \cdot \frac{\left(\frac{NG_{m,pancreas}(t)}{V_{m,pancreas}}\right)^2}{EC50_I{}^2 + \left(\frac{NG_{m,pancreas}(t)}{V_{m,pancreas}}\right)^2} + Q\frac{NI_{m,liver}(t)}{V_{m,liver}} - Q\frac{NI_{m,pancreas}(t)}{V_{m,pancreas}} \; (mIU/h) \qquad \text{(S 6)}$$

$EC50_I$: Glucose concentration resulting in half-of-maximum response to insulin (mM/L)

The insulin secretion capacity per unit volume of β cell is given by:

$$\sigma(t) = \sigma_{max} \cdot \left(1 - \frac{t^2}{\alpha \quad + t^2}\right) (mIU/L/h) \qquad \text{(S 7)}$$

σ_{max}: Maximal insulin secretion capacity per unit volume of β cell (i.e. at the start of the co-culture) (mIU/L/h)

α : Parameter defining the sigmoidal dependence on time of $\sigma(t)$ (h^2)

The variable $V_{\beta,islets}(t)$ (L) describes the changes in volume of β-cells in the pancreatic islets over the co-culture time, according to the following equation:

$$\frac{dV_{\beta,islets}(t)}{dt} = k_v(-d_0 + r_1 G_{slow,pancreas}(t) - r_2 G_{slow,pancreas}(t)^2) \cdot V_{\beta,islets}(t) \;\; (L/h)$$

where d_0 is the death rate at zero glucose (h^{-1}) and $r_1 = r_{1,r} + r_{1,a}$ (L/mmol/h) and $r_2 = r_{2,r} + r_{2,a}$ ($L^2/mmol^2/h$), where $r_{1,r}$, $r_{1,a}$ (L/mmol/h), $r_{2,r}$, $r_{2,a}$ ($L^2/mmol^2/h$) are parameters that determine the dependence of the replication and apoptosis rates on glucose. The parameter k_v was introduced to account for potential differences in behaviour between pancreatic islets in our *in vitro* system and rodent islets the model of Topp et al. (1).

The variable $G_{slow,pancreas}(t)$ (mmol/L) represents the long-term average (i.e daily) glucose concentration in the co-culture mediumas given by:

$$\frac{dG_{slow,pancreas}(t)}{dt} = \frac{G_{pancreas}(t) - G_{slow,pancreas}(t)}{\tau_{slow}} \; (mmol/L/h)$$

(S 8)

$G_{pancreas}(t)$: Glucose concentration in the pancreas compartment (mmol/L)

τ_{slow} : Time constant that determines the averaging of $G_{pancreas}(t)$ over time (h)

The concentrations of glucose and insulin in each compartment were calculated by dividing the insulin and glucose content, respectively, by the volume of co-culture medium in the compartment:

$G_{liver}(t) = \frac{NG_{m,liver}(t)}{V_{m,liver}}$ (mmol/L)

(S 9)

$G_{pancreas}(t) = \frac{NG_{m,pancreas}(t)}{V_{m,pancreas}}$ (mmol/L)

(S 10)

$I_{liver}(t) = \frac{NI_{m,liver}(t)}{V_{m,liver}}$ (mIU/L)

(S 11)

$I_{pancreas}(t) = \frac{NI_{m,pancreas}(t)}{V_{m,pancreas}}$ (mIU/L)

(S 12)

Glucose and insulin samples in the MPS were obtained by pooling samples from both the liver and the pancreas compartment. Therefore, the resulting glucose and insulin measurements ($G(t)$ and $I(t)$, respectively), were computed as:

$$G(t) = {G_{liver}(t) \cdot V_{sample,liver} + G_{pancreas}(t) \cdot V_{sample,pancreas}} \Big/ {(V_{sample,liver} + V_{sample,pancreas})} \text{ (mmol/L)} \quad \text{(S 13)}$$

$$I(t) = {I_{liver}(t) \cdot V_{sample,liver} + I_{pancreas}(t) \cdot V_{sample,pancreas}} \Big/ {(V_{sample,liver} + V_{sample,pancreas})} \text{ (mIU/L)} \quad \text{(S 14)}$$

where $V_{sample,liver}$ and $V_{sample,pancreas}$ are the volumes of co-culture media collected from the liver and pancreas compartment in each sample (15 µl).

The initial conditions for the model states are listed below:

$NG_{m,liver}(0) = (G_{dose} + \Delta G_{d1}) \cdot V_{m,liver}$ (mmol)

$NG_{m,pancreas}(0) = (G_{dose} + \Delta G_{d1}) \cdot V_{m,islets}$(mmol)

$NI_{m,liver}(0) = \Delta I_{d1} \cdot V_{m,liver}$ (mIU)

$NI_{m,pancreas}(0) = \Delta I_{d1} \cdot V_{m,pancreas}$ (mIU)

$t(0) = 0$ (h)

$G_{int}(0) = 0$ (mmol·h/L)

$G_{slow,pancreas}(0) = 5.5$ (mmol/L)

$V_{\beta,islets}(0) = 8.8 \cdot 10^{-9}$ (L)

where ΔG_{d1} (mmol/L), ΔI_{d1} (mIU/L) are offset parameters that account for experimental errors related to the media exchange performed at day 1. The experimental errors in the glucose concentration can be due to, for instance, variations in the volume of co-culture medium or added glucose when the glucose media is performed. Non-zero values of insulin concentration at t=0 might be explained by co-culture medium remaining in the chip (both in the culture compartments and the microfluidic channel) during the medium exchange corresponding to the first GTT. Similarly, the parameters $(\Delta G_{d13}, \Delta I_{d13})$ were included in the model to account for errors in concentrations during the media exchanges performed at day 13. Across our historical experimental data, we did not find systematic errors in glucose values at the beginning of the culture between single-liver and liver-islet cultures, and therefore they were modelled with an offset parameter.

2. Comparison of hepatic insulin elimination rate constants in the HepaRG liver-islet MPS with human *in situ* values

We hypothesized that impaired insulin clearance by the HepaRG/HHSteC spheroids contributes to the enhanced insulin levels in the HepaRG liver-islet MPS compared to human *in situ* values. To address this question we investigated, based on our scaling approach, whether the translated value of insulin

clearance for Experiment 1 was lower than the one found in humans. The estimated value of $CL_{i,spheroids}$ which represents the insulin clearance rate normalized by the total volume of HepaRG cells in the HepaRG/HHSteC spheroids, was translated to a corresponding human value based on Eq. S5 and the values of translated parameters in Table 2:

$$CL_{human} = \frac{CL_{I,spheroids} \cdot (V_{HepaRG,spheroids})_{human}}{(V_{m,liver})_{human}} \; (1/h)$$

where $CL_{I,spheroids}$ is the *in vitro* (MPS) value listed in Table 2 ($CL_{I,spheroids}$=17.81(1/h)) $(V_{HepaRG,spheroids})_{human}$ and $(V_{m,liver})_{human}$ are the translated human values in Table 2 ($(V_{HepaRG,spheroids})_{human}$=0.34 (L) and $(V_{m,liver})_{human}$=1.5 (L)). The resulting CL_{human} value is 4.04 (1/h). We compared this value with the hepatic insulin elimination rate constant reported by Dalla Man et al. (2), $m_3(0) = 17.09$ (1/h), finding the following ratio between these values:

$$\frac{m_3(0)}{CL_{human}} = \frac{17.09}{4.04} = 4.23$$

We translated the insulin elimination rate constants measured experimentally in the MPS (S4 Fig) to the corresponding human values as follows. First, we calculated the value of $CL_{I,spheroids,exp}$ from the experiments using the estimated value of k and Eq. S5:

$$CL_{I,spheroids,exp} = \frac{k \cdot V_{m,liver}}{V_{HepaRG,spheroids}}$$

The resulting values of $CL_{I,spheroids,\mathbf{exp}}$ were 2.12 and 1.85 (1/h) for days 1 and 6, respectively. These values were then translated to human using the approach described previously, and the calculated values $CL_{human,exp}$ were 0.48 and 0.42 (1/h). Therefore, the hepatic insulin elimination rate constants measured in the MPS were approximately 35 times smaller than those reported in humans. The experimental $CL_{I,spheroids,exp}$ values are within the range of model-based estimated $CL_{I,spheroids}$ values for the experiments considered in the study.

We also measured insulin binding in an experiment with empty organoid compartments to investigate insulin absorption/adsorption in the MPS and ensure that our measurements of insulin clearance were not overestimated. We performed a 7-day experiment with media exchanges every 24 hours. In each media exchange, a specific amount of insulin was added to the culture medium. Samples of the culture media were taken directly after each media exchange (t = 0 h) as well as 24 hours after (t = 24 h). The measured insulin concentrations (S5 Fig) were relatively stable between medium exchanges and after incubation in the chips, suggesting that insulin adsorption/absorption can be neglected in our MPS.

References

1. Topp B, Promislow K, Devries G, Miura RM, Finegood DT. A model of β-cell mass, insulin, and glucose kinetics: Pathways to diabetes. J Theor Biol. 2000;206(4):605–19.

2. Man CD, Rizza RA, Cobelli C. Meal Simulation Model of the Glucose-Insulin System. IEEE Trans Biomed Eng. 2007;54(10):1740–9.
