# Supplementary material for: Integrated experimental-computational analysis of a HepaRG liver-islet microphysiological system for human-centric diabetes research
Source: PLoS Comput Biol. 2022 Oct 19;18(10):e1010587. doi: 10.1371/journal.pcbi.1010587 (PMC9621595; doi:10.1371/journal.pcbi.1010587)
Supplement: Attachment — Submitted filename: Response to reviewers.pdf [file pcbi.1010587.s010.pdf]

Author's response to reviews of

"Integrated experimental-computational analysis of a liver-islet microphysiological system for human-centric diabetes research"

PLOS Computational Biology, PCOMPBIOL-D-22-00202

RC: Reviewer's comment AR: Author's response Line number

Reviewer #1

The manuscript "Integrated experimental-computational analysis of a liver-islet microphysiological system for human-centric diabetes research" describes an interesting advancement in the modelling and interpretation of microphysiological systems. The specific focus on T2DM is highly relevant and generally the work is well carried out. The authors should clarify some details to improve the presentation further.

AR: We thank the reviewer for the positive assessment of our manuscript. We address the comments and clarify the details below.

RC1: (Introduction) Please describe interspecies differences for the specific disease - T2DM - to further motivate the need for MPS in this case

AR: We have expanded the introduction on page 4, lines 79-85 in the clean, revised manuscript, to describe interspecies differences between humans and rodent models of T2DM that are relevant for our study.

RC2: (Introduction) The sentence "By integrating 3D cultures of human organ-specific cells in a 87 microfluidic platform, these in vitro systems aim to recreate key microenvironmental aspects of in vivo 88 tissues (flow, multicellular architectures, and tissue-tissue interfaces), thereby being more 89 physiologically relevant than standard cell cultures (9–11). " should be changed as many (most) MPS are not 3D cultures

AR: We are grateful to the reviewer for pointing out our mistake here, and have modified this sentence accordingly (page 4, line 92)

RC3: Clearly define what is one experiment? Is that one system? Where the some of the seven systems run at the same time? There are some explanations in different parts of the MS, but still the replicates vs experiments are unclear.

AR: One experiment corresponds to one donor of pancreatic islets and involves several Chip2 chips, each of them comprising two platform replicates. Across all experiments, the number of replicates used in each experiment was 5 ± 2. In each experiment, the time-series measurements were performed on each replicate and combined to calculate the mean and variability (SEM) reported in the manuscript. We have clarified this in the methods section (page 6, lines 157-160), as well as in Figures 1, 4, 5 and 6.

RC4: In section 2.2.1 "The parameter (mIU/L/h) represents the maximal insulin secretion rate of the sigma_max cells (i.e. at the beginning of the co-culture). The parameter alpha is estimated based on the experimental measurements. For large values of this parameter, the decrease in insulin secretion capacity over time would be negligible." Describe how you experimentally derive alpha?

AR: The parameter α is among the 12 parameters that are estimated through optimization against the experimental measurements. This parameter is estimated for each experiment (i.e. on an experiment-specific basis). We have clarified this in the methods section of the manuscript (page 12, lines 316-317). The estimated experiment-specific values for this parameter are listed in Supplementary Tab. S3. One can note that the value of this parameter correlates with the decrease in insulin secretion observed in the data for each of the experiments. For instance, the experiments with the largest and lowest decrease in insulin secretion (Experiments 4 and 6 in Supplementary Fig. S2, respectively) correspond to the lowest and largest values of α in Supplementary Tab. S3.

RC5: In fig 4, 6 and 7 – give a metrics for the correlation instead of stating acceptable or good

AR: In the revised version of the manuscript, we have provided the value of the cost function in relation to the threshold value from the χ^2 test to quantify the agreement for Figs. 4 and 6 (page 15, lines 412-414 and page 17, line 491). For the prediction in Figure 7, there is good visual agreement with the experimental data, in terms of temporal evolution and glucose and higher insulin levels in the pancreas compartment in comparison to the liver compartment. However, the model predicted insulin concentrations in the liver compartment between days 5 and 9 which are larger than those found experimentally, and the visual agreement is not supported by the statistical χ^2 test. Nevertheless, given that the model is not calibrated using these data, there is still a good agreement between the model prediction and the experimental data, and the differences between predictions and data are comparable to the size of the experimental variability. We have clarified this in the revised version of the manuscript (page 18, lines 507-515)

RC6: The hypothesis that the pancreatic islets have abnormal insulin secretion due to prolonged exposure to hyperglycemic conditions should be verified with explained experimental conditions

AR: Indeed, testing this hypothesis experimentally would be very interesting. While we already provide data showing that GSIS values of pancreatic islets that have been cultured under hyperglycemia are larger than for islets that have been cultured under normo- and hypoglycemia (Figure S1), establishing a comparison of this measure with humans is non-trivial. Therefore, since we proposed two main hypotheses for increased insulin concentrations in the MPS (enhanced insulin secretion by the pancreatic islets and/or decreased insulin clearance by the liver spheroids), we decided to instead test the second hypothesis experimentally. Since several studies have provided values of insulin clearance rates in humans, it would be easier to find a comparison with the MPS values in this case. We performed an independent, 7-day, single-liver experiment to measure hepatic insulin clearance experimentally. We found that this clearance is indeed lower in the MPS than in humans, and we have therefore modified the translation approach to account for this effect. We have expanded the results (page 19, lines 547-561, and Fig. 8) and the discussion sections of the manuscript (pages 23 and 24, lines 705-722) to address this, as well as the Supplementary (Section 2).

RC7: In discussion please keep the references format

AR: We are grateful to the reviewer for pointing out this mistake and have modified these references in the discussion accordingly (page 20, line 580 and page 21, line 642)

RC8: If I understand correctly only HepRG cells were used and not primary hepatocytes – add a discussion how this affect the outcome and in vivo translation of the work

AR: The HepaRG cell line used in our study has been shown to display several features relevant to primary human hepatocytes in terms of gene expression and proteins involved in glucose and lipid metabolism, as well as functional insulin responsiveness. In the current study, the glucose and lipid metabolism are of high importance, and previous reports have described gene expression, functional regulatory pathways, and functional machinery for glucose and lipid metabolism in HepaRG cells, with high similarity to primary human hepatocytes[1–3]. However, this cell line also has some limitations that have been recognized in previous studies related to drug metabolism and toxicity, and similar limitations might as well be applicable to our study and the proposed *in vivo* translation. A possible limitation of the HepaRG cell line is that it has a low expression of the glucagon receptor, which might result in glucagon insensitivity[2]. This study is, however, based on monolayer cultures with only HepaRG cells. Our 3D co-culture model might have improved glucagon sensitivity, similar to the improved insulin sensitivity in comparison to monocultures that we have reported previously[4].

Comparing the functionality of *in vitro* HepaRG and primary human hepatocytes (PHHs) to *in vivo* liver function is challenging, especially since the operating and co-culture conditions in our system can differ from other studies (e.g. medium composition, 3D vs. 2D cultures, circulating flow vs. static cultures). When applying the model-based translation, we show that is possible to translate the MPS responses to human to a certain extent, suggesting the suitability of HepaRG cells for this purpose. The use of PHHs could improve the physiological relevance of our model and should be investigated in future studies, but it may involve other limitations. Translatability issues could arise in this case depending on the PHHs culture model. For instance, PHHs have been shown to loose responsiveness to glucagon and insulin stimulation in conventional monolayer cultures after only three days of culture[5].

We have expanded the discussion section in the revised manuscript to address these potential limitations of using HepaRG cells instead of primary human hepatocytes (page 22, line 648-667)

RC9: The discussion is interesting but a bit too long and general in the end - I suggest that the authors focus on the implications of the specific study The work would be strengthened by a short conclusion section at the end.

AR: We have shortened the final part of the discussion and included a short conclusion section (page 25, lines 751-760) in the revised manuscript.

Reviewer #2

In the presented paper, Casas and colleagues utilize a microfluidic system with recirculating medium in which two cell compartments containing "liver cells" and human islets are serially connected. This system is then used to derive a mathematical model that can be used to analyze liver-islet crosstalk in vivo. This goal is worthwhile and highly ambitious. Unfortunately however, the manuscript has multiple, at least in part, severe limitations that curb my enthusiasm about this work. Most importantly, as detailed below, the physiological relevance of the model is very limited due to the simplistic fluidic circuit and the use of hepatoma cells instead of primary hepatocytes. Furthermore, the experimental validation is very sparse. For instance, the authors make broad predictions about insulin resistance, islet cell death, insulin secretion rates etc. without having measured any of these parameters, despite the fact that it should be easily possible.

AR: We want to thank the reviewer for this initial assessment, which we to a large extent agree with: it is an ambitious goal, and this current manuscript is only to be considered as a first step, and not as a final ready-to-use product. In other words, the current work is a first step in a

systematic process to build and fine-tune a model to be able to translate MPS data to human *in vivo*, with the long-term goal of replacing certain animal experiments with *in vitro* MPS experiments plus computational after-analysis. Before this becomes possible, much additional fine-tuning and validation of both the experimental and computational model must be done. Such fine-tuning may include for instance the replacement of HepaRG with PHHs, but also extension of the system with additional organs to make the glucose consumption/clearance mechanisms more realistic, adding other readouts or treatments such as glucagon and GLP-1, etc. Concerning model validation, we are not claiming that our assumptions are perfect and that the proposed model is the only one that can explain data. However, the model is mechanistically based and is – in our view – already a reasonably sound model that at least can explain current data, and that shows promise to be useful in translating to human. Finally, in the modelling process, it is natural to iteratively test different likely factors by introducing them into the model and later design experiments to validate/reject them.

RC1: The authors state that they "previously presented a two-organ MPS integrating liver and pancreas", which they now use for further experiments and modeling approaches. However, the authors fail to state that this model uses a hepatic cell line (HepaRG), which cannot serve as a faithful model for the liver as an organ. This fact is not stated in either abstract or introduction and it appears to this reviewer that this is purposefully done in an attempt to mislead the reader regarding the physiological relevance of the utilized model. It is critical that it is made unambiguously clear in title, abstract and introduction that the study uses a hepatoma cell line and not primary liver cells. For instance, "HepaRG" spheroids should be used throughout the manuscript instead of "liver spheroids".

AR: We have explicitly included HepaRG in the abstract and introduction, and clearly stated that the term liver spheroids corresponds to HepaRG spheroids (page 6, lines 164, 165).

RC2: In relation to the comment above, IVIVE from cancer cells to human in vivo values seems highly problematic. How can transformed cells be used to study "healthy" conditions?

AR: The HepaRG cell line used in our study has been shown to display several features relevant to primary human hepatocytes in terms of gene expression and proteins involved in glucose and lipid metabolism, as well as functional insulin responsiveness. In the current study, the glucose and lipid metabolism is of high importance, and previous reports have described gene expression, functional regulatory pathways, and functional machinery for glucose and lipid metabolism in HepaRG cells, with high similarity to primary human hepatocytes[1–3]. However, this cell line also has some limitations that have been recognized in previous studies related to drug metabolism and toxicity, and similar limitations might as well be applicable to our study and the proposed *in vivo* translation. A possible limitation of the HepaRG cell line is that it has a low expression of the glucagon receptor, which might result in glucagon insensitivity[2]. That study is, however, based on monolayer cultures with only HepaRG cells. Our 3D co-culture model might have improved glucagon sensitivity, similar to the improved insulin sensitivity in comparison to monocultures that we have reported previously[4].

Comparing the functionality of *in vitro* HepaRG and primary human hepatocytes (PHHs) to *in vivo* liver function is challenging, especially since the operating and co-culture conditions in our system can differ from other studies (e.g. medium composition, 3D vs. 2D cultures, circulating flow vs. static cultures). When applying the model-based translation, we show that is possible to translate the MPS responses to human to a certain extent, suggesting the suitability of HepaRG cells for this purpose. The use of PHHs could improve the physiological relevance of our model and should be investigated in future studies, but it may involve other limitations. Translatability issues could arise in this case depending on the pHHs culture

model. For instance, PHH have been shown to lose responsiveness to glucagon and insulin stimulation in conventional monolayer cultures after only three days of culture[5]

We have expanded the discussion section in the revised manuscript to address these potential limitations of using HepaRG cells instead of primary human hepatocytes (page 22, line 648-667)

RC3: Again, related to the overstatement of results and its physiological relevance: the model essentially recirculates medium through a linear connection of HepaRG spheroids and islets, thereby neglecting other tissues that play the predominant role in glucose clearance (skeletal muscle, adipose tissue, CNS). Similarly, there is no/very limited clearance of insulin. Moreover, medium is exchanged every 48h resulting in highly unphysiological, step-wise changes of insulin/glucose levels.

AR: We acknowledge the current limitations of the MPS model, and our intention with this manuscript is to exemplify how these limitations can be partially overcome with computational modelling, as some of them might be particularly challenging to address experimentally (due to constraints in design and experimental conditions). In the original manuscript we pointed out some of these limitations, generally throughout the manuscript and explicitly in the discussion section (page 23, lines 686-689), where we mentioned some of the most relevant limitations (slower kinetics, properties of the co-culture medium and the organoids, missing glucose consuming organs and signaling mechanisms, imbalanced media-to-tissue ratio in comparison to humans). In the revised manuscript, we have expanded this paragraph to be more specific about the limitations suggested by the reviewer.

We thank the reviewer for pointing out the possibility of having very limited clearance of insulin in the MPS. We had hypothesized this as a cause for having increased insulin levels after translation of the responses to humans. We have now further investigated this hypothesis by performing a new single-liver *in vitro* experiment to estimate the hepatic insulin clearance in our system. We have expanded the results (page 19, lines 547-560, and Fig. 8) and the discussion sections of the manuscript (pages 23 and 24, lines 705-722) to address this, as well as the Supplementary (Section 2).

Regarding the media exchanges every 48 hours, this is chosen to have ample time to see changes in glucose concentration since, as pointed out previously, the glucose kinetics are slower in the MPS compared to *in vivo*. Another contributing factor is the practical handling of the two-organ MPS. To have medium changes mimicking three meals a day is currently not feasible with manual handling of the chips. However, a robotic system is under development, which in the future would allow for more frequent medium changes. Furthermore, our understanding of the system (by modelling) does not necessarily have to be based on data from perfectly physiologically realistic input signals. In general, many different system perturbations, for instance in the form of various glucose inputs, can help us to unravel the system and therefore we believe that the current set-up is relevant.

RC4: No information is provided regarding other important pancreatic endocrine signaling molecules, such as glucagon. This should be added.

AR: We thank the reviewer for this relevant suggestion. Indeed, it would be relevant to study the behavior of other pancreatic endocrine signaling molecules, such as glucagon, in the system, and its effect on glucose homeostasis. In our experimental protocol, we decided to focus only on glucose and insulin and try to understand these two variables first. The main reason was that the sampling volume we can take from the chip at each time point is very limited and to get stable readouts of glucose and insulin we did not have much volume left for other analyses (for instance, glucagon ELISA requires 10 µL). While out of the scope of this study, it would be good to look at pancreatic endocrine signaling molecules in follow-up

experiments to further improve the mathematical model and our understanding of the system. We have explicitly addressed this point in the revised discussion section of the manuscript (page 22, lines 645-647).

RC5: The setup raises the question about the added value of such a simple perfusion system. Would there be differences compared to static co-culture of HepaRG spheroids and islets in the same well of e.g. a conventional 384-well plate?

AR: We have performed parallel experiments with only HepaRG spheroids or pancreatic islets in both static cultures and in the MPS settings and see improved functionality in the MPS co-cultures. We have however not performed static co-cultures since by placing islets and HepaRG spheroids in the same well there is a risk that these organoids merge together. This could potentially affect the functionality of the organoids, and hamper further use of the cells in posterior analyses, such as GSIS or gene expression analysis.

RC6: More information should be provided for the pancreatic islets: Were native islets used or were islets dissociated and reaggregated? Does the model only include endocrine cells or also exocrine cells.

AR: We are grateful to the reviewer for pointing out this. The pancreatic islet microtissues were dissociated and reaggregated from primary islets and include only endocrine cells ($\boldsymbol{\alpha}$, $\boldsymbol{\beta}$ and $\boldsymbol{\delta}$ cells). We have clarified this in the Methods section (page 7, lines 192-194).

RC7: Scaling factor of liver and islets: If I understand correctly, the authors scale HepaRG cells : islet cells (which should also include alpha and delta cells; possibly also exocrine cells) as 40 : 1. However, the human pancreas has in toto around 1 billion b cells, compared to around 200 billion hepatocytes. Thus, scaling liver:islet seems off by at least a factor of 5. Furthermore, as stated above all the other hundreds of billions of cells in the human body that also take up glucose and insulin are entirely neglected.

AR: We scaled HepaRG liver spheroids : pancreatic islets as 40:10, and the downscaling from human was based on the total number of hepatocytes and beta cells for liver and pancreas, respectively. This scaling is in the order of 100.000 for both organs, but when estimating the scaling liver : islet based on literature data this ratio is, as indicated by the reviewer, not exactly one (although the scaling is still in the order of 100.000 for both organs).

Considering the number of pancreatic islets in the MPS (10) and the number of cells per islet (~2000[6]), of which ~50% are β cells[6], we estimate a total of 10.000 β cells in the MPS. Therefore, assuming that the human pancreas has 1 billion β cells in total, the scaling factor is 100.000. Regarding scaling of the liver organoid, it comprises 40 HepaRG liver spheroids with approximately 24000 HepaRG cells and 1000 stellate cells each[4], resulting in a million cells in total. Assuming that the human liver comprises 200 billion hepatocytes, this leads to a scaling factor of 200.000. We have modified the manuscript to clarify that the downscaling factor is in the order of 100.000 rather than exactly 100.000 (page 8, line 203).

Nevertheless, given that we use the computational model to scale the MPS to human, this mismatch in the individual scaling of the organoids is not as critical as it would be without computational modelling. In fact, a number of approaches have been proposed to scale organ-on-a-chip platforms. These approaches include not only direct scaling, which is the one implemented in our study, but also allometric scaling[7], scaling based on the residence time of blood in each organ[8], and functional scaling[9]. In all these approaches, identical scaling factors for all organs is not a requirement for achieving physiological responses. We have shown that our computational model combines several of these approaches, by accounting for the scaling of the organs and the residence times.

Regarding the lack of other glucose consuming cells in our MPS, we have addressed this in the revised manuscript at several points throughout the introduction (page 6, lines 137-139) and discussion sections (page 23, lines 687-689).

RC8: The authors culture their cells in medium containing 860nM insulin. Before transfer, the cells were washed twice and then transferred to insulin-free medium. Given the huge excess of insulin in the pre-culture medium compared to physiological concentrations (around 2nM), more information should be provided regarding the dilution factors of the washed. Furthermore, insulin concentrations should be measured at t0 after transfer to make sure that there was no considerable carry-over and insulin levels are indeed close to zero.

AR: We aim at removing insulin from the pre-culture medium completely. Therefore, we wash the liver spheroids twice with 1 ml PBS/0.1% BSA and incubate the spheroids for 2 hours in 1 mL chip medium without insulin. During this pre-incubation, insulin remaining in the spheroids after the wash steps has time to diffuse out of the spheroids. Before the addition of PBS/0.1% BSA and medium, we remove as much liquid from the wells as possible. However, a small volume of the medium remains around the liver spheroids. Even if we estimate that this remaining volume is around 50 μL (which is likely an overestimation of the volume that typically remains), the dilution factor of the wash and pre-incubation step is together around 8.000 resulting in a dilution of the 860 nM to 0.1 nM insulin. Finally, the spheroids are transferred from the pre-incubation well to the multi-organ-chip (600 μL) by collecting them with a wide-bore tip and letting them gravitate from the tip into the culture compartment. In this step, almost no medium should be transferred. If we however estimate that around 10 μL are transferred (which we again think is overestimated), the 0.1 nM insulin will be further diluted by a factor of 60 to 0.002 nM insulin. Furthermore, the glucose tolerance test is not initiated on the day of organ model transfer but only on the next day (day 1) after a complete medium exchange. Therefore, we do not think that insulin from the pre-culture would appear during the glucose tolerance test.

Regarding the concentration at t0 after transfer (t=0) we have measured this in several experiments (Experiment 1, Fig. S2, Experiment 2, Figs. 4,7, Experiment 5, Fig. S2, Experiment 6, Fig. S2, Experiment 7, Fig. S2). Furthermore, the computational model includes a parameter to account for this potential offset in insulin at t=0 (Supplementary material, page 16, lines 212-213). One can observe that the insulin concentrations at t=0 are close to zero and have low values in comparison to the peak insulin value at GTT d1-3. We hypothesize that this could be due co-culture medium remaining in the chip (both in the culture compartments and the microfluidic channel) during the medium exchange corresponding to the first GTT. We have clarified this in the revised manuscript. (Supplementary, page 16, lines 215-217)

RC9: Model parameters: The rate of beta-cell death was obtained from the literature. However, this is likely highly dependent on the model/platform/culture conditions and should thus be measured in the actual device.

AR: As the reviewer suggests, it would be interesting to perform measurements on beta-cell apoptosis and proliferation in our MPS. For one of the experiments, we performed cell proliferation analysis by EdU incorporation, but there were several limitations in translating these measurements to apoptosis and proliferation rates for beta-cells in our computational model. These limitations include the need for optical clearing to image all cells in intact islets, and the inability to measure proliferation and apoptosis exclusively in beta cells. We further elaborate on these limitations in the paragraph below.

We performed beta-cell proliferation analysis by staining of islets followed by imaging. We tested different labeling and detection stains (4 last days in chip in the presence of EdU for

proliferation detection, YO-PRO-1, Annexin V and PI staining post-chip for apoptosis) in intact islets. While, in general, these stains penetrate efficiently through the islets, detection by fluorescence microscopy is challenging. Although ‘optical sectioning’ (stacking images taken at increasing height from bottom of islet upwards, to create a 3D picture) is possible with laser scanning two-photon microscopy, spinning disk confocal or light-sheet approaches, the scatter of light in heterogeneous tissue (by e.g. ribosomes, nuclei, mitochondria, lipid droplets, membranes, ECM) hinders imaging in cell spheroids. It is not possible to image the inner part of the islet, but only the outer layers. Imaging of the cells in the middle of each islet is only possible after fixing and optical clearing of the islet to equilibrate the refractive index and thereby reduce inhomogeneities in light scatter. Fixing the islets was problematic for measuring apoptosis, since when the cells are fixed they are dead so all cells stain. This approach, however, allowed us to measure proliferation, but unfortunately it was not possible to distinguish between proliferation of beta-cells and other cell types. We therefore tried to identify beta-cells with insulin antibodies, but while EdU labeling is nuclear and clearly separated between cells, insulin is cytosolic and cell-cell boundaries are difficult to see in a 3D cell spheroid, and therefore this approach turned unsuccessful.

We therefore estimated the rate of beta-cell death by estimating a scaling factor for the literature values, via optimization against the experimental measurements. The reason for introducing a scaling factor was to maintain the qualitative behavior of beta-cell replication and apoptosis, which we expect to be similar to that reported in literature[10]. Based on our GSIS data, shown in Fig. S1, and assuming that beta-cell apoptosis and proliferation is driven by long-term glucose levels, we hypothesize that at hyperglycemia (11 mM), the difference between replication and apoptosis rates will be larger than at normo- and hypoglycemia (5.6 and 2.8 mM, respectively). This behavior is consistent with the bell-shaped behavior of beta-cell mass reported by Topp et al.[10]. We acknowledge that, quantitatively, these replication and apoptosis rates will be dependent on the model/platform/culture conditions and therefore introduce the scaling factor k_v, which is estimated on an experiment-specific basis. We have clarified this in the manuscript (page 12, lines 316-317)

Besides the mentioned technical difficulties, we emphasize the importance of experimentally measuring beta-cell proliferation and beta-cell volumes to compare against the model-based values in the discussion section of the revised manuscript (page 21, lines 622-631)

RC10: What is the material of the MPS? Did the authors ever measure insulin absorption/adsorption to the plastic of the culture device. If not, this should be measured in order to not overestimate insulin clearance.

AR: The MPS material is a mixture of glass, plastic and PDMS. It is correct that especially for PDMS, absorption/adsorption can be problematic, for example for small molecules. In our experience, however, this is not the case for proteins in the TissUse HUMIMIC multi-organ-chips as the PDMS surface is modified and pre-treated in order to reduce protein adsorption. Nevertheless, we have measured insulin binding in empty chips (no spheroids) by replacing the medium in the chips on day 0, 1, 2, 3, 4, 5 and 6 with medium to which we added a specific amount of insulin. We took samples directly after medium addition (t=0) as well as after 24 hours (t = 24h). Looking at the results in the figure underneath, the insulin concentrations are fairly stable between medium addition and after incubation in the chips. Therefore, we concluded that binding of insulin does not seem to be an issue in our system. We have also discussed the lower amounts of insulin from day 0-1 and day 1-2, as these could be interpreted as rapid initial insulin binding. However, as we would expect a further decline of the insulin concentration between t=0 and t=24h if binding was the reason for the lower concentrations on day 1 and 2, we believe that the lower insulin concentrations are more likely caused by errors during the dilution of the insulin stock and its addition to the medium which was freshly prepared every day. We have discussed insulin absorption/adsorption in the revised version of the manuscript (Supplementary Material, page 17, lines 245-252, and page 7, Fig. S5)

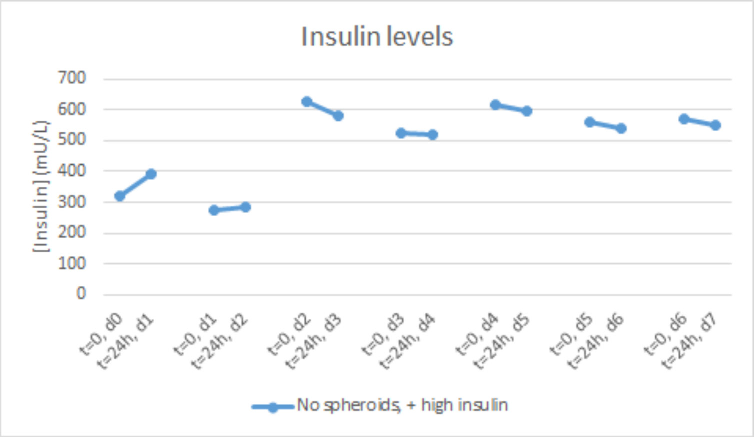

RC11: Promotional company material should not be cited (InSphero, 2016).

AR: We are grateful to the author for pointing out this mistake. We have included a citation to a recent publication instead (page 13, line 347)

RC12: Figure 4A-B: Why is the glucose concentration in the co-culture already at t0 lower than in the monoculture (around 9mM compared to 11mM in the HepaRG-only setup)? This is particularly apparent as the only cycles with measured data (the first and the last) in the co-culture setup start at 9mM, whereas all cycles in between start at 11mM.

AR: We believe the difference in starting values is not due to the fact that one experiment is a co-culture while the other one is single-liver, but rather measurement errors which may have different causes, such as variations in the actual volume of co-culture media, glucose doses and the accuracy of the glucose measurements. These errors are accounted for as offsets in the computational model. We have clarified this in the revised version of the manuscript (Supplementary, page 16, lines 213-215).

RC13: Figure 4E: The authors conclude that "insulin sensitivity in the liver spheroids decreases progressively as they are exposed to hyperglycemic periods during the co-culture (Fig. 4E). This decline in insulin sensitivity leads to reduced glucose utilization by the liver spheroids, resulting in higher daily glucose levels over time." – However, the model uncertainty does not allow to draw conclusions about increase in insulin resistance and follow-up conclusions are questionable. Rather, insulin sensitivity should be measured directly, e.g. by pAKT Western blotting.

AR: We agree with the reviewer that performing further insulin sensitivity readouts like AKT phosphorylation would be highly interesting to investigate the hypotheses implemented in the mathematical model. This is currently under investigation and will be part of a future manuscript. Nevertheless, with this manuscript we wanted to propose mechanistic hypotheses that can describe our experimental data, being insulin resistance induced by hyperglycemia one of them. We have therefore reformulated the quoted paragraph to clarify that the insulin resistance curve is a prediction of the mathematical model, and therefore solely a possible explanation for the observed glucose and insulin responses.

RC14: Figure 5A, 6A: The experimental GTT data (the last cycle) shows no difference between hypo-, normo- and hyperglycemia, thus essentially showing that 2w culture in

different glucose levels do not alter insulin sensitivity or that the range of insulin concentrations covered in the device (0.3 to 2.5nM) is not sufficient to alter glucose uptake in HepaRG cells, likely because cells were relatively insulin resistant (due to preculture in 800nM insulin) to begin with.

AR: There is indeed little difference between glucose concentrations for the different glycemic regimes at day 13. The computational model used in this study represents the hypothesis that each of these glycemic regimes will lead to different degrees of insulin sensitivity, which in turn will yield similar glucose consumption patterns when exposed to the different insulin concentrations in the media corresponding to these glycemic regimes. Liver spheroids under hyperglycemia will exhibit the highest degree of insulin resistance, but are also exposed to the highest insulin concentration levels amongst the different glycemic regimes. This leads to similar glucose consumption as in hyper- and normo-glycemia, as in this case the liver spheroids will develop little to no insulin resistance, but the insulin concentrations will also be lower in this case. When implementing this computational model we find an acceptable agreement with the experimental data based on statistical analysis, but this does not imply that this is the only biological mechanisms responsible for this behavior.

As the reviewer suggests, there might be other hypotheses for this observed behavior in glucose consumption for varying glycemic regimes. Analyzing these hypotheses would require an in-depth model-based hypothesis testing[11], which would involve extensive modelling work to create computational models for each hypothesis, as well as additional experiments to validate or reject hypotheses. This is outside the scope of the current work, but could be investigated in further studies. We have expanded the discussion section of the revised manuscript to address these different hypotheses (page 24, lines 721-724)

RC15: Figure 7: The measured values in 7A, B, D and E do not fit the model trends. For instance, there does not seem to be a decrease in insulin concentrations over time (7D-E), whereas there seems to be an increase in glucose levels, which is not predicted by the model (7A-B).

AR: The model does predict an increase in glucose levels that agrees well with the measurements from the pancreas compartment, but slightly underestimates the glucose concentration at t=288h in the liver compartment. However, this experimental value of glucose might as well be underestimated due to the experimental reasons previously mentioned, and indeed the glucose concentrations in both compartments should be comparable given the slow glucose dynamics and the recirculation time in the MPS. The model did however predict larger insulin concentrations than those found experimentally between days 5 and 9. Nevertheless, given that the model is not calibrated using these data, there is still a good agreement between the model predictions and the experimental data, and the differences between predictions and data are comparable to the size of the experimental variability. We have clarified this in the revised version of the manuscript (page 18, lines 507-515)

Reviewer #3

It took me a while to understand what the authors were trying to accomplish with this paper, but in the end I was mostly convinced that the work is correct and worthwhile. The goal was to use modeling to establish a microfluidic pseudo-tissue preparation (called MPS) containing human pancreatic islets and liver spheroids and allowing for inter-organ communication as an intermediate platform between in vitro and in vivo approaches. The preparation studied here was previously introduced in Ref. 12 but without modeling. In the present paper, the model of Topp (Ref. #41) for diabetes pathogenesis is applied to the MPS but with significant modification to account for the different glucose-insulin dynamics compared to humans and rodents. The MPS is rich enough to exhibit insulin resistance and impaired insulin secretion in response to

hyperglycemia, though these differ from how those phenomena manifest in vivo, as detailed below. I think the MPS is a long way from being usable as a model for type 2 diabetes, but it may be a good first step in that direction.

AR: We thank the reviewer for this fair assessment, which we agree with: the current addition of computational modelling to the MPS-system is a first step (but only a first step) in an important direction.

General comments:

RC1: Whereas the Topp model treats insulin resistance as an external forcing factor, implicitly representing consequences of obesity and likely representing resistance mostly in muscle and adipose tissue, the current model looks at insulin resistance in the liver spheroids, attributed to hyperglycemia. It doesn't seem likely that this plays an important role in human diabetes, but it appears to be involved in the MPS.

AR: We thank the reviewer for this interesting comment. We are, in this work, primarily studying the MPS system, where hyperglycemia does indeed seem to be involved in the insulin resistance, at least to some extent. We have added a comment in the discussion, pointing out that the mechanisms for insulin resistance in the MPS system may differ from those in humans (page 21, lines 643-644).

RC2: Insulin-mediated glucose uptake (IMGU) plays a central role in the Topp model, but liver glucose transport does not depend on insulin, Instead, IMGU here represents an increase in diffusion of glucose into the liver as intracellular glucose is converted to glycogen (p. 9). This process is very slow: restoration of basal glucose after a glucose challenge takes 48 hours, in contrast to the 2 hours in vivo (Fig. 4A, text l. 624). This seems to me to result mainly from the lack of muscle tissue in the MPS, and I was surprised that this was not mentioned in the paragraph beginning at l. 615. Please comment.

AR: While the lack of muscle tissue in the MPS will certainly lead to slower glucose consumption compared to *in vivo* we believe that, assuming that the liver is responsible for approximately 45% of the total postprandial glucose uptake in humans, this is probably not the main cause for the slow glucose kinetics in the MPS. Adding muscle would only increase glucose uptake by approximately a factor of two, and would probably not account for the factor required (a factor in the order of 10). Instead, we believe that the main cause for this is the difference in scaling between MPS and humans (volumes of organoids, co-culture medium and flow rate).

Indeed, by scaling the volumes of the organs and residence times to human proportions, we can account for the differences in temporal dynamics between MPS and humans. To illustrate this, we include below simulations of the translation to human where we have not accounted for other glucose-consuming organs (the multiplying factor in insulin-independent glucose disposal rate ($\boldsymbol{E_{G0}}$) and insulin sensitivity ($\boldsymbol{S_I}$) is set to 1, instead to 2.22 as specified in line 534). Also, the insulin secretion capacity has not been decreased. The insulin clearance elimination rate constant was modified as described in lines 552-555. One can note that after these changes, the dynamics of glucose consumption are much faster than in the MPS and comparable to humans, despite us not having added the other organs. Furthermore, Lee et al.[12] reported results from a two-organ MPS including pancreas and muscle, and glucose consumption was also very slow, with an approximate decrease from 17 to 15 mM in three hours of co-culture.

In the revised manuscript, we have pointed out the lack of muscle tissue as a contributing, but probably not dominating, cause to the slow glucose dynamics in the MPS (page 23, lines 686-689)

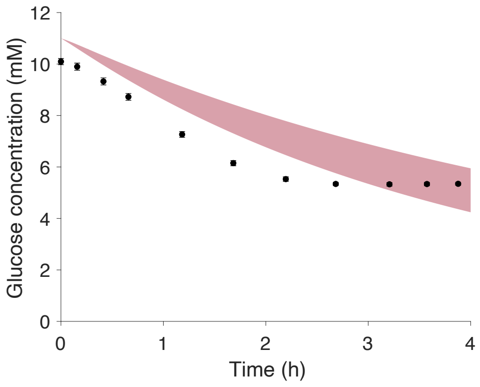

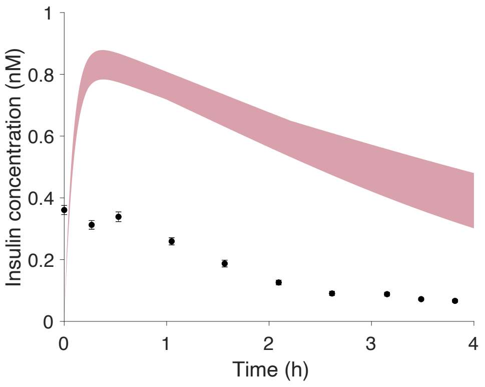

RC3: (a) One advance over the Topp model is to make insulin secretion capacity (sigma) time dependent. A priori, it is difficult or impossible to separately identify sigma and beta-cell mass (here, volume) since they appear multiplicatively in Eq. 2, but it works here because the parameters for mass/volume are fixed at the values in Topp's initial paper (l. 319 - 322, Table 1). Please comment explicitly on this point. I would not call these values "the established range" (l. 452); the Topp model was qualitative and meant to illustrate the key dynamical features, not fit to data.

AR: We chose to include a time-decreasing insulin secretion capacity because the decrease in insulin secretion levels over time seemed too large to be caused by beta-cell apoptosis exclusively. For example, in Experiment 1, the maximal insulin concentration decreased by approximately 75 % between the GTTs performed at day 1 and day 7 (Fig. S2). This decrease is larger than the apoptotic rates defined in the Topp model, but also than those reported in studies with beta-cells *in vitro*[13]. While it is difficult to draw conclusions about the beta-cells in our system from other studies that involve different experimental conditions, we hypothesize that the decrease in insulin secretion is not only due to changes in beta cell apoptosis and proliferation rates, but also due to decreased function of the beta cells (for instance due to the composition of the co-culture medium). We chose to model this as a time-dependent insulin secretion function, and the fact that the model provides a statistically acceptable agreement with the data indicates that it is a plausible explanation for the behavior in our system. However, as the reviewer indicates, it is not possible to separately identify the insulin secretion capacity (sigma) and beta-cell mass, and there may be other possible mechanistic hypotheses that provide good agreement with the data. Given that this is a first study integrating a mathematical model with the MPS, we have focused on what we considered key hypotheses rather than aiming for completeness and including many possible ones. We have emphasized the possibility of additional mechanisms for beta-cell dynamics in the discussion section of the revised manuscript (page 21, lines 625-631)

Regarding the parameters in the Topp model[14] for beta-cell dynamics, we chose the same parameter values as in their study, but included a parameter k_v to account for possible differences with our experimental setting. In practice, this implies that we set the same stable points as in Topp et al[14], but different values of the proliferation/apoptosis rates. Ideally, we would like to estimate the parameters in the beta-cell dynamics model using experimental measurements from our MPS, but we found several limitations in translating these measurements to apoptosis and proliferation rates for beta-cells in our computational model (please see our response under Reviewer 2, Comment RC9). We did, however, perform GSIS

experimental measurements (Fig. S1) that showed increased insulin secretion for 11 mM compared to 5.5 mM, and for 5.5 mM compared to 2.8 mM. These differences could be explained by different proliferation/apoptosis rates between the glycemic regimes, and we adopted the values of the stable points in the Topp model since these can describe what we observe experimentally. In the revised manuscript, we have rewritten "the established range" as "the range suggested by Topp et al." (page 17, line 473-474).

(b) The model assumes that sigma is a decreasing function of time (Eq. 3), and it does in fact decrease markedly over the 13-day study. However, the formulation precludes the possibility that, like mass, sigma could also show a compensatory increase. Please comment on this modeling choice.

AR: This is a very good suggestion and it would indeed be interesting to study this hypothesis in our model. For instance, Ha et al.[15] proposed a glucose-dependent enhancement of beta-cell function via a reduction in the number of ATP-sensitive K^+ (KATP) channels in the plasma membrane of the beta-cell. As explained in our previous response, based on the values of beta-cell apoptosis rates that we would expect from previous *in vitro* studies[13], we hypothesize that it's more likely that beta-cell function decreases over time, but there might be several mechanisms involved, and also at different time-scales (the behavior described by Ha et al.[15] corresponds to a short-term compensation). It would be interesting to measure beta-cell proliferation and apoptosis in our system, and possibly measurements of glucose and insulin at additional time points to capture additional behaviors that we might have not fully characterized in our current set-up. We have discussed this additional hypothesis in the revised version of the manuscript (page 21, lines 625-631)

RC4: Related to point #2: I was impressed but also perplexed by the success of the upscaling to the human in vivo case. The model showed reasonably good agreement, but required doubling of insulin- and non-insulin mediated glucose uptake (to account for the absence of muscle tissue in the MPS), increased insulin clearance (not sure why this was needed) and decreased insulin secretion capacity (no justification was offered for this, other than that it improved the fit). It is further suggested that including endogenous production would have improved the agreement (l. 528).

My enthusiasm was dampened by the number of ad hoc adjustments (though most were plausible), and my perplexity stems from my belief that accounting for muscle is not just a quantitative matter, but involves qualitatively different signaling pathways (insulin-dependent GLUT4). That left me wondering whether the good agreement was fortuitous and whether the envisioned application to drug development for humans is credible. Please comment on this.

AR: We would like to thank the reviewer for these insightful comments. The success of the upscaling may seem perplexing, but it is – in fact – quite intuitive. Intuitive ballpark calculations are as follows. An evident problem with glucose and insulin dynamics is that glucose uptake is too slow in the MPS: it takes 48h instead of approximately 2 to restore basal glucose. At the same time, the media-to-tissue ratio is also larger in the MPS compared to humans (in the order of 100:1 in the MPS vs. 1:4 in humans[16]). Therefore, the glucose added to the medium in each medium exchange in the MPS, which corresponds to adding the meal to the intestines/blood in humans, is added to a larger pool in comparisons to humans. Therefore, given the same rate of glucose uptake, it will take longer to deplete the larger pool. It is therefore natural and intuitive that the glucose uptake is much slower in the MPS. Apart from these volume differences, there are also differences in residence times for both liver and pancreas between the MPS and humans, given the relation between flow rate in the circulating medium and the volume of circulating medium, which has an effect on glucose and insulin concentrations.

A second major difference between the responses in the MPS and humans pertains to the insulin concentration. This is a difference in concentrations, and therefore is not as trivial as the previous difference in temporal dynamics. The difference has to be an actual difference in rates, i.e. either a difference in secretion and/or elimination of insulin. Following the reviewer's comment, we have performed additional experiments to characterize insulin clearance in the MPS, and further quantified and clarified how much of the insulin differences are likely to stem from altered secretion and clearance in the revised version of the manuscript. We have expanded the results (page 19, lines 547-560, and Fig. 8) and the discussion sections of the manuscript (pages 23 and 24, lines 705-722) to address this, as well as the Supplementary (Section 2).

Regarding the different signaling pathways, our current approach to account for other organs is based on the assumption that the liver accounts for 45 % of the total postprandial glucose uptake (both insulin-dependent and independent). This approximation is certainty a first step towards accounting for missing organs, rather than a final solution. Unfortunately, as indicated in the manuscript (pages 21 and 22), we currently lack measurements of intracellular fluxes to allow us to characterize the intracellular pathways for the current organs. Adding new organoids to the MPS is a substantial effort that requires optimization of the co-culture medium to accommodate each new organ. It will indeed be highly interesting to include intracellular pathways, also from currently missing organs, in our translation to human, but this is outside the scope of the present study.

Specific comments:

RC5: l. 72 - 73: "over time they are unable to meet the increased insulin demand and T2DM manifests": It would be better to say "they may be" instead of "they are" because in most cases the beta cells are able to successfully compensate for insulin resistance.

AR: We are grateful to the reviewer for pointing out this mistake. We have made the corresponding change in the revised manuscript.

RC6: l. 397: "Tab. S3" should be "Tab. S2".

AR: This mistake has been corrected in the revised manuscript

RC7: l.398 - 400: "as opposed to the slower glucose consumption after 13 days": Please quantify this, as it is difficult to evaluate by eye.

AR: We have included the exact glucose concentration values 8 hours after the start of the GTTs at day 1 and day 13 in the revised manuscript (page 15, lines 415-418).

RC8: p. 15, top: Please say something about Fig. 4D.

AR: We thank the reviewer for pointing this out. We have commented in Fig. 4D in the revised version of the manuscript (page 15, lines 425-426).

RC9: p. 16, description of Fig. 5: why is there no panel for sigma, in contrast to Fig. 4?

AR: We did not include a panel for sigma initially since it would be the same for the two glycemic conditions represented in the plot, and we intended to highlight the differences between conditions. We have included a panel for sigma in the revised manuscript for clarity (Fig.5).

RC10: p. 17, description of Fig. 6: please acknowledge that even though the predictions of glucose and insulin were validated, there is no obvious way to validate the predictions about insulin resistance, beta-cell volume, or secretion capacity.

AR: We are grateful to the reviewer for pointing this out. We have included this statement in the revised manuscript (page 17, lines 491-494).

RC11: l. 511 - 512: the model results in Fig. S3 were described as "in agreement" with those in humans. This is confusing and possibly misleading because the point of Fig. S3 was to say that without the adjustment in secretion capacity, the agreement was poor.

This statement referred to the temporal dynamics of the response exclusively, which are indeed in agreement with the temporal dynamics in humans although the magnitude of the response was not. We have reformulated this sentence in the revised manuscript to make it clearer.

RC12: l. 582: "These predictions agree with the previously established hypothesis": Rather than "agree with" I would say "follow from" so as not to give the impression that the predictions are in agreement with some measurement.

AR: We are grateful to the reviewer for pointing this out, and we have rewritten this sentence in the revised version of the manuscript.

References

1. Caron, S. *et al.* Farnesoid X receptor inhibits the transcriptional activity of carbohydrate response element binding protein in human hepatocytes. *Mol. Cell. Biol.* 33, 2202–2211 (2013).

2. Samanez, C. H. *et al.* The human hepatocyte cell lines IHH and HepaRG: models to study glucose, lipid and lipoprotein metabolism. *Arch. Physiol. Biochem.* 118, 102–111 (2012).

3. Tascher, G. *et al.* In-Depth Proteome Analysis Highlights HepaRG Cells as a Versatile Cell System Surrogate for Primary Human Hepatocytes. *Cells* 8, 192 (2019).

4. Bauer, S. *et al.* Functional coupling of human pancreatic islets and liver spheroids on-a-chip: Towards a novel human ex vivo type 2 diabetes model. *Sci. Rep.* 7, 14620 (2017).

5. Davidson, M. D., Lehrer, M. & Khetani, S. R. Hormone and Drug-Mediated Modulation of Glucose Metabolism in a Microscale Model of the Human Liver. *Tissue Eng. - Part C Methods* 21, 716–725 (2015).

6. De Gaetano, A. *et al.* Mathematical models of diabetes progression. *Am. J. Physiol. Metab.* 295, E1462–E1479 (2008).

7. Wikswo, J. P. Scaling and systems biology for integrating multiple organs-on-a-chip. 13, 3496–3511 (2014).

8. Sung, J. H., Kam, C. & Shuler, M. L. A microfluidic device for a pharmacokinetic-pharmacodynamic (PK-PD) model on a chip. *Lab Chip* 10, 446–455 (2010).

9. Maass, C., Griffith, L. G., Cirit, M. & Stokes, C. L. Multi-functional scaling methodology for translational pharmacokinetic and pharmacodynamic applications using integrated microphysiological systems (MPS). *Integr. Biol.* 9, 290–302 (2017).

10. Topp, B., Promislow, K., Devries, G., Miura, R. M. & Finegood, D. T. A model of β-cell mass, insulin, and glucose kinetics: Pathways to diabetes. *J. Theor. Biol.* 206, 605–619 (2000).

11. Cedersund, G. Conclusions via unique predictions obtained despite unidentifiability – new definitions and a general method. *FEBS J.* 279, 3513–3527 (2012).

12. Lee, D. W., Lee, S. H., Choi, N. & Sung, J. H. Construction of pancreas–muscle–liver microphysiological system (MPS) for reproducing glucose metabolism. *Biotechnol. Bioeng.* 116, 3433– 3445 (2019).

13. Maedler, K. *et al.* Glucose Induces β-Cell Apoptosis Via Upregulation of the Fas Receptor in Human Islets. *Diabetes* 50, 1683 LP – 1690 (2001).

14. Topp, B. G., Atkinson, L. L. & Finegood, D. T. Dynamics of insulin sensitivity, β-cell function, and β-cell mass during the development of diabetes in fa/fa rats. *Am. J. Physiol. Metab.* 293, E1730–E1735 (2007).

15. Ha, J., Sherman, A. S. & Satin, L. S. A Mathematical Model of the Pathogenesis, Prevention, and Reversal of Type 2 Diabetes. *Endocrinology* 157, 624–635 (2015).

16. Wagner, I. *et al.* A dynamic multi-organ-chip for long-term cultivation and substance testing proven by 3D human liver and skin tissue co-culture. *Lab Chip* 13, 3538–3547 (2013).
