# Supplementary material for: Integrated experimental-computational analysis of a HepaRG liver-islet microphysiological system for human-centric diabetes research
Source: PLoS Comput Biol. 2022 Oct 19;18(10):e1010587. doi: 10.1371/journal.pcbi.1010587 (PMC9621595; doi:10.1371/journal.pcbi.1010587)
Supplement: Attachment — Submitted filename: Response to reviewers.pdf [file pcbi.1010587.s011.pdf]

Author's response to reviews of

"Integrated experimental-computational analysis of a liver-islet microphysiological system for human-centric diabetes research"

PLOS Computational Biology, PCOMPBIOL-D-22-00202R1

RC: Reviewer's comment AR: Author's response Line number

<u>Reviewer #2</u>

The authors have addressed some of my comments very well. However some important concerns remain:

RC1: While some of the overstatements have been removed, multiple instances that could mislead readers remain. The use of a hepatoma cell line cannot qualify as a model of a liver organ and as such the model is clearly not microphysiological, as it lacks anatomical hepatic structures, other hepatic cells, gradients of oxygen and other important hepatic functions etc. As such, I reiterate that the model should not be called a "liver-islet microphysiological system". Furthermore, the term "liver spheroids" should be avoided as this term is used in the literature for models of primary human liver cells. Please rephrase to "HepaRG spheroids".

AR: Following the reviewer's suggestion, we have rephrased the term "liver spheroids" in the revised version of the manuscript. We have rephrased it to ""HepaRG/HHSteC spheroids" to indicate that the spheroids include both HepaRG and human hepatic stellate cells. Regarding the comment on referring to the *in vitro* model as "liver-islet microphysiological system", we have considered the reviewer's suggestion and referred to it as "HepaRG liver-islet microphysiological system". Given the broader scope of the journal, which is not limited to *in vitro* tissue and organ models, we are concerned that renaming it as HepaRG-pancreas microphysiological system" might not be informative enough to some of the journal's audience. We have rephrased these terms accordingly in the manuscript title, throughout the text, and in the figures (Fig. 2) and figure legends (Figs. 2, 4, 5 and 8) and Supplementary methods and figures.

RC2: I follow the authors rationale that at least one purpose of the manuscript is to show that a mathematical model might overcome certain limitations of the system that arise from technical issues, such as the lack of models of other glucose sink tissues. This was not clear to me after the first reading and I thank the authors for this clarification. However, the quantification of insulin sensitivity/resistance of HepaRG cells using pAKT Western blot should be conducted as I am concerned that these cells are highly insulin resistant already at the start of the experiment due to long-term culture of cell lines at micromolar insulin concentrations while passaging. However, if those cells do not respond to physiological insulin levels as they should, the system can hardly model in vivo response kinetics.

AR: We thank the reviewer for this suggestion. It is indeed relevant to investigate whether HepaRG/HHSteC spheroids are insulin sensitive at the beginning of the co-culture. We had performed an experiment where we measured AKT phosphorylation following the pre-culture period (at micromolar insulin concentrations), and prior to their transfer to the MPS to start the co-culture. The results, included in our previous publication (Bauer et al., 2017) are shown in the figure below. As shown in the figure, we found a significant difference in Akt phosphorylation between insulin-stimulated and unstimulated cells (white bars and black bars, respectively) in cultures of HepaRG/HHSteC spheroids. We have clarified that the HepaRG/HHSteC spheroids are sensitive to insulin in the beginning of the co-culture in the revised version of the manuscript (page 8, lines 220-222). The method for quantifying insulin sensitivity using pAKT measurement is described in detail in https://www.nature.com/articles/s41598-017-14815-w.

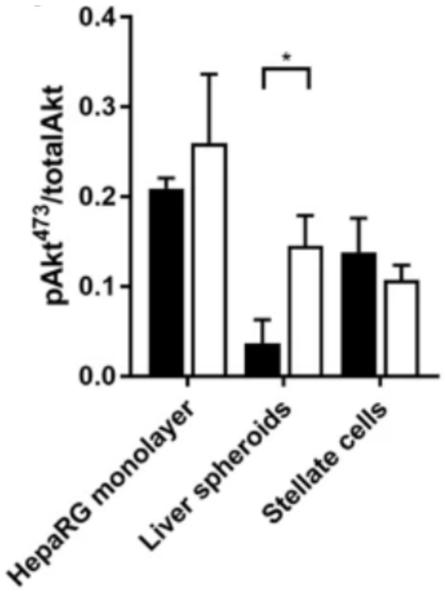

Figure 1: Quantification of insulin sensitivity of HepaRG/HHSteC spheroids prior to the co-culture, following the pre-culture period. Phosphorylated AKT (pAKT, serine-473) normalized to total AKT in unstimulated (black bars) and insulin-stimulated (1 nM, white bars) conditions. *P < 0.05 using un an unpaired, two-tailed Student t-test. Data shown as mean + SD (n = 3). Adapted from (Bauer et al., 2017).

RC3: Regarding my previous comment 12: I accept that pipetting errors might indeed accommodate for these differences; however, if all measured values in co-culture are lower than in monoculture, the source of this apparently systematic error should be investigated and not be corrected by simply modeling a different offset.

AR: We thank the author for this interesting insight, which we have investigated. We evaluated our historical data for experiments including both single-liver and liver-islet cultures, and found the following offsets for two additional experiments (referred to as **9-day experiment A, 9-day experiment B** in the table below). Only the offsets at day 1 and day 7 are included, since these experiments correspond to 9-day cultures instead of 15 days, this being the reason why they were not included in the manuscript. Contrary to the experiment mentioned by the reviewer (**Experiment 2**),

in these experiments the glucose offsets were larger in the co-culture compared to single-liver. Therefore, we cannot conclude based on our data that the glucose offsets are systematically higher in single-liver cultures compared to co-cultures. We have clarified this in the revised version of the manuscript (Supplementary material, page 16, lines 221-223).

**Experiment**	**G day1-3, t=0 (mM)**		**G day 7-9, t=0 (mM)**	
	Single-Liver	*Co-culture*	*Single-liver*	*Co-culture*
9-day experiment A	10.82 ± 1.19	14.38 ± 1.7	10.07 ± 0.02	10.23 ± 0.1
9-day experiment B	11.2 ± 2.48	11.2 ± 2.63	9.7 ± 0.58	10.73 ± 1.71

Reviewer #3

RC1: I am satisfied with the responses to the points raised in my first review and I feel the authors have been straightforward in acknowledging the limitations of the preparation as well as its potential as a first step toward improved systems. I endorse in particular the response to Reviewer #2, RC 14: it is reasonable that glucose doesn't rise given the increase in insulin secretion.

One minor comment about the added text on p. 23 on whether beta-cell function rises or falls in response to insulin resistance. It may first rise and then fall, which would be consistent with the observations in animal and human studies that insulin levels first rise and then fall.

AR: We thank the reviewer for this insightful comment, which we have addressed in the revised version of the manuscript (page 24, lines 691-693).
